# Integrating the SD-CLUE-S and InVEST models into assessment of oasis carbon storage in northwestern China

**DOI:** 10.1371/journal.pone.0172494

**Published:** 2017-02-23

**Authors:** Youjia Liang, Lijun Liu, Jiejun Huang

**Affiliations:** 1 School of Resources and Environmental Engineering, Wuhan University of Technology, Wuhan, Hubei Province, China; 2 Meteorological Service Center of Gansu Province, Lanzhou, Gansu Province, China; Pacific Northwest National Laboratory, UNITED STATES

## Abstract

Spatio-temporal integrated assessment of land-use change impacts on carbon storage services is a new and important research field in land science and landscape ecology. The objective of this paper is to use an integrated SD-CLUE-S and InVEST model to simulate and predict land-use changes impacts during 2000–2018 on carbon storage at pixel and regional scales in the Zhangye oasis, Northwest China. The SD-CLUE-S model was used to simulate land-use change, and three land-use scenarios (current trend, moderate protection, and strict protection) were defined in collaboration with oasis socioeconomic development and ecological environment conservation by local government. The InVEST model was then used to simulate land-use change impacts on carbon storage at different scales in the oasis. The results showed that: (1) the effects of built-up land expansion were especially notable, with a rapid decrease in cropland during 2009–2018; (2) the strict protection scenario saved the largest amount of carbon storage for the oasis compared with the current trend and moderate protection scenarios. The scientific value of this study has been to show that the proposed modeling method can be used to reflect different land-use patterns and their effects on ecosystem services at multiple scales in the oasis. Furthermore, this research can be used to help government managers encourage stakeholders to contribute funds and strategies to maintain oasis landscape patterns and ecological processes by implementing local plans for potential conservation projects.

## Introduction

Carbon storage is a widely used indicator of ecosystem services because it measures the responsiveness of productive capacity and ecological resilience to changes in terrestrial ecosystems [1–2]. Land-use intensification is a major part of land-use change and has become a primary cause of ecosystem service depletion [3–5]. To improve the sustainability of ecosystem services and socioeconomic development around desert-oasis ecosystems, it is important to consider the significant impacts of land-use change under human activities on the dynamic balance of carbon storage due to built-up land growth and vegetation degradation, which in turn have threatened the delivery of multiple ecosystem services in desert-oasis ecosystems.

Despite advances in the development of decision-making and analytical frameworks for ecosystem service assessment [6–8], the various patterns of land-use change are an underlying concern at the regional scale because the effects of land conversion and management practices can cause significant impacts on ecosystem services and products [9–10]. Like many regions of northwestern China, the Zhangye desert oasis is a relatively data-poor region. A few plot-scale studies have focused on quantitative assessments of vegetation change on oasis ecological processes at the regional scale [11–13], but little quantitative modeling has been carried out on carbon storage processes and their relationship with land-use change in the rapidly developing oasis. Information on carbon storage services derived from field survey data and quantitative models can improve decision-making for land management and socioeconomic planning by local governments. The impacts of various socioeconomic development practices on the resulting land-use and carbon storage services and the effects of scenario-based land-use change have been less studied in the oasis. Integration methods and case studies of carbon storage services into land management planning are still lacking [14]. To address these issues, integrated modeling methods are becoming useful because they can simulate and predict land-use impacts on carbon storage services at multiple scales [15].

In this study, the method used to determine the spatial distribution and approximate magnitude of carbon storage was the InVEST 3.3.0 (Integrated Valuation of Environmental Services and Tradeoffs) model, which was developed by the Natural Capital Project [16–17]. The InVEST model is a spatially explicit modeling tool that can evaluate the impact of different land uses on multiple ecosystem services [18]. However, the lack of land-use simulation models to integrate with the InVEST and with user-defined preference assumptions may limit model application in ecosystem service research. For example, the InVEST model does not generate different scenarios of land-use patterns to select the optimal one that minimizes negative impacts across ecosystem services, nor does it consider the potential impacts of land restoration [19]. Therefore, methods to integrate land-use models and InVEST for ecosystem service assessment need to be developed.

The CLUE-S (Conversion of Land Use and its Effects at Small regional extent) model was developed as a GIS-based modelling tool based on an empirical analysis of location suitability combined with simulation of competition and interactions among the spatio-temporal dynamics of multiple land-use processes [20–21]. However, the model’s ability to represent the macro-demands of land use based on a given scenario is weak. For this reason, SD-CLUE-S was developed as a land-use model by integrating CLUE-S with a system dynamics (SD) model [22]. The model was used to simulate both top-down and bottom-up drivers of land-use change processes in a spatially explicit modeling method that can enhance the accuracy of simulated results.

In this paper, the primary objective was to assess the effects of historical land-use patterns and alternative land-use change scenarios on carbon storage services by modeling and mapping with SD-SCLUE-S and InVEST in the Zhangye oasis, Northwest China. This study aims to predict land use in the oasis using a combination of multiple socioeconomic development plans, ecological and environmental conservation, urban expansion, and oasis agricultural practices. In addition, this quantitative assessment was achieved by simulating the spatial distribution of and changes in carbon storage to obtain a better understanding of the consequences of ecosystem service and land-use change. The main objectives of this paper were achieved as follows: (1) based on land-use maps for 2000, 2005, and 2009 and other auxiliary data for the oasis, land-use change was simulated and validated using the SD-SCLUE-S model from 2000 to 2009; (2) three scenarios were developed to simulate future land-use change, which represent different combined strategies and policies for land supply-demand balance from 2009 to 2018 in the oasis; (3) the variation of carbon storage from 2000 to 2009 was quantified using an InVEST model that was locally influenced and relevant to policy-makers and local stakeholders; and (4) the potential impacts of future land-use change on oasis carbon storage were also studied using various 2009–2018 land-use scenarios.

## Methods

### Study area

**Fig 1:**
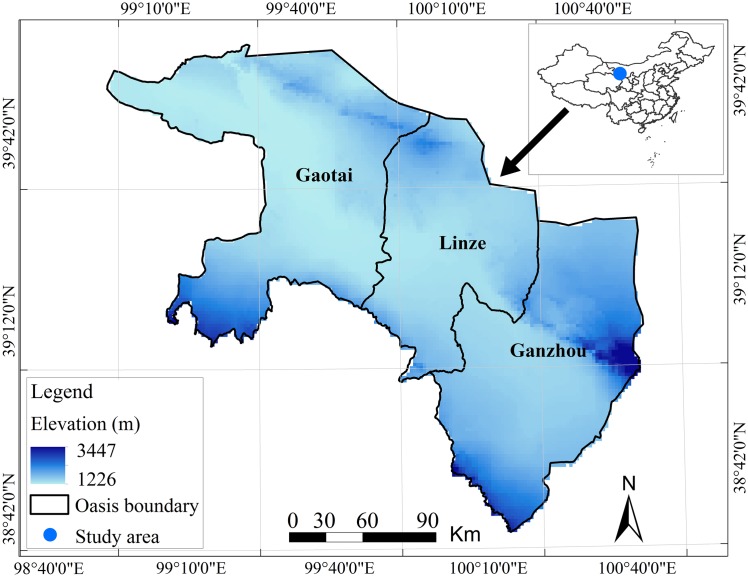
Location of Zhangye oasis.

The Zhangye oasis is located in the central part of the Heihe River Basin, between 98°57′E and 100°52′E and between 38°32′N and 39°42′N, and has been suffering from serious water scarcity and a sharp decline of wetland resources (Fig 1). The total oasis area is about 1.13×10^4^ km^2^. Annual precipitation in the study area varies from 62 to 156 mm, and annual evaporation is 1000–2000 mm. The elevation in this oasis ranges from 2000 m to 1340 m.

Three counties are located in the oasis, one of which is a county-level city (Ganzhou) and two of which are counties of Zhangye City (Linze and Gaotai). All these counties are examples of typical oasis regions. In 2000–2009, the total population of this oasis increased by 77.94%, from 352,380 to 627,022, the Gross Domestic Product (GDP) increased by 95.99%, from 4.06×10^10^ RMB to 7.96×10^10^ RMB, and the urbanization rate increased by 43.77% (1 USD = 6.09 RMB) [23–24]. In the study area, natural oases have been gradually replaced by artificial oases, driven by changes in human activities and water resources as well as interactions between these two key factors over the past 2000 years [25–26]. However, rapid urbanization of the oasis has been a feature of the past 10 years. Increasing population pressure and economic growth have inevitably resulted in excessive use of water resources and ecosystem degradation in the oasis.

### Data

A number of source datasets were combined to assess carbon storage services in this study. Land-use maps of the oasis for 2000, 2005, and 2009 were collected from the Environmental and Ecological Science Data Center for West China (EESDCWC, http://westdc.westgis.ac.cn). These maps were derived from remote-sensing Landsat Thematic Mapper (TM) images using a standard interpretation procedure. The maps were categorized into different types before land-use change simulation according to the Second National Land Survey (http://www.gov.cn/jrzg/, S1 Table). Six typical land-use types were then identified in this study, including built-up land, woodland, cropland, grassland, water, and unused land. The overall classification accuracies of these maps were 86.53%, 86.73%, and 85.80% in 2000, 2005, and 2009 respectively [27].

Soil data were used to represent the potential limiting factor for regional development based on different soil components and were obtained from the China Dataset of Soil Properties for Land Surface Modeling (CDSP-LSM) [28]. To represent the proportion of coarse sand and gravel over 50% of the grid cells in the study area, the attribute value was set to 100 to represent a restricted region, and other attribute grid cell values was set to 0 to represent non-restricted regions for land-use expansion. The annual average Normal Differential Vegetation Index (NDVI) for 1998–2008 was calculated from the Long-Term Vegetation Index Dataset of China: SPOT Vegetation NDVI, and the initial dataset was obtained from EESDCWC. Digital elevation model (DEM) data were obtained from the Digital Heihe River site (http://heihe.westgis.ac.cn/) at the original resolution of 100 m. Socioeconomic data were obtained from the Zhangye Statistical Yearbook for 2000–2009 [23–24].

A number of ancillary datasets for integrated modeling were also collected from the EESDCWC, including irrigation canals, transportation networks, city centers, and administrative boundaries. All the map data were completely preprocessed and uniformly projected into a Universal Transverse Mercator projection with 100-m resolution in ArcGIS 10.0, which was a sufficient scale to reflect detailed information on oasis land-use change [29].

### SD-CLUE-S model

A land allocation module (mainly local) and a land-use demand module (mainly regional) were included in the SD-CLUE-S model [22]. In the land allocation module, the CLUE-S model was used to simulate the spatial dynamics and spatial allocation of land-use types [30–31]. The total number of converted grid cells was constrained by the predictable results from the land-use demand module. The transition probabilities of different grid cells were determined simultaneously by their land demand, spatial policy restrictions, spatial location characteristics, and conversion parameter settings [22,31]. Specifically, the spatial distributions of the six land-use types were quantified using a binomial logistic regression equation with the percentages of the types as the dependent variables and the driving factors of NDVI (*X*_*1*_), soil (*X*_*2*_), elevation (*X*_*3*_), slope (*X*_*4*_), and transportation (*X*_*5*_) as independent variables. The five driving factors were selected based on their suitability, availability, and stability of data. Transportation was used to calculate the distance to the nearest transportation network, and the other four driving factors were used to describe the natural environmental conditions. Finally, the conversion probability of a certain grid cell to a specific land-use type was defined by using the following logistic model:

$$\text{ln}\left(\frac{P_{m}}{1-P_{m}}\right)=b_{0}+b_{1}X_{1,m}+\;b_{2}X_{2,m}\ldots \ldots +b_{k}X_{k,m}\;$$ (1)

where *P*_*m*_ is the probability of a specific grid cell being occupied by the *m*-th land-use type, *X*_*k*,*m*_ indicates the *k*-th driving factor of the *m*-th land-use type, and *b*_*k*_ is the weighting coefficient of the *k*-th driving factor. Once the probability *P*_*m*_ for a given grid cell *(i*,*j)* of the *m*-th land-use type has been calculated, the spatial change in this land-use type can be simulated during a given time period. The land conversion process terminates when the total area of the *m*-th land-use type satisfies the demand as estimated by the land demand module.

The demands for different land uses are determined by socioeconomic factors in this integrated modeling framework. At the regional scale, SD models have been widely used to predict land-use demand during a given period because they are useful for analyzing the complex connections between regional socioeconomic development and land use and can also be used to express temporal consequences based on different socioeconomic development scenarios [22,32–33]. Therefore, a parameterized SD model was used to estimate the demand for different land-use types as a whole with specific scenarios of socioeconomic development, including 33 parameters in this preliminary study [22]. The parameterized SD model was developed in the system dynamics software STELLA 8.0.

**Fig 2:**
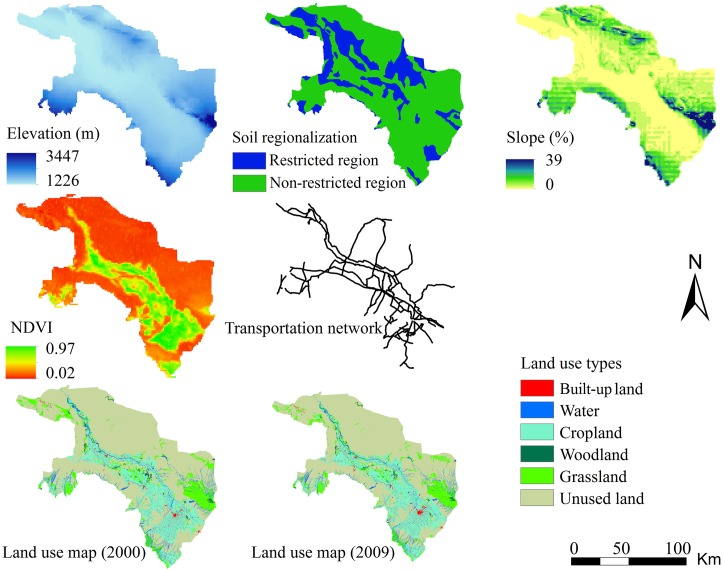
General input and validation data for the SD-CLUE-S model.

To ensure the reliability of the simulated land-use change results, calibration and validation of the SD-CLUE-S model were carried out based on the input data from 2000 to 2009. The general input variables of the SD-CLUE-S model are shown in Fig 2. The land-use map of the Zhangye oasis in 2000 was used as an input seed layer to predict land-use patterns from 2000 to 2009. As described in detail in an earlier study [34], the annual incoming flow upstream of the oasis has remained at a higher rate while annual precipitation has decreased since 2005. This evidence indicates that changing water resources have significantly affected spatial land-use patterns, leading to a change in the oasis scale and to adjustment of crop-planting structures since 2005. For this reason, the actual land-use map in 2005 was then used to calibrate the performance of the SD-CLUE-S model. The Kappa index was used as a standard measurement to verify the overall simulation accuracy of the land-use model because it is recognized as an effective indicator of accuracy for land classifications [2,35]. The best combination of coefficients (Table 1) with the optimal Kappa index was selected for scenario simulation. The actual land-use map in 2009 was used to validate the accuracy of the calibrated SD-CLUE-S model. The Kappa values (*p*<0.01) of different oasis land types in 2009 were in the following order: cropland (0.8723) > unused land (0.8541) > built-up land (0.8203) > water (0.8109) > grassland (0.7731) > woodland (0.7605). The calculation of Kappa index was completed in the statistical software SPSS 19.0.

**Table 1:** Calibrated weighting coefficients for the SD-CLUES-S model in the study area (significance of all variables at *p*<0.01).

Variable	NDVI	Soil	Elevation	Slope	Transportation	Constant
Cropland (km^2^)	1.0203	0.0102	0.0023	−0.0161	−0.0013	7.2465
Woodland (km^2^)	2.1427	1.1744	0.0034	0.0887	−0.1043	−9.8617
Grassland (km^2^)	1.6562	0.5869	0.0042	0.0405	0.0021	-9.6972
Water (km^2^)					−0.0012	0.3181
Built-up land (km^2^)			−0.0035	−0.2625	1.0002	0.4027
Unused land (km^2^)	0.0542	0.1626	0.0027	0.0024	0.0101	3.4378

### Scenario design and prediction

After SD-CLUE-S was calibrated and validated successfully, the model was then used to predict future land-use change in Zhangye oasis up to 2018. SD-CLUE-S provides a spatio-temporal simulation tool for exploring the potential consequences of land management policies adopted by local decision-makers. The integrated modeling method can be used to define various land-use demand scenarios based on historical input data to simulate likely areal coverages in future years. To demonstrate the usefulness of the integrated modeling method and to provide a comprehensible context to oasis managers, three scenarios were simulated in the study area, including (1) a historical land-use demand scenario (S1) reflecting land demand growth as a continuation of the historical period; (2) a moderate protection scenario (S2) in which land demand growth was limited by setting critical indicators of economic and social development; and (3) a strict protection scenario (S3) in which land demand growth was simulated with multiple strict protections in the study area (Table 2). These scenarios were rooted in local development and decision-making in the oasis in the past few years. The main controlling factors of land-use patterns in the oasis were derived from local socioeconomic development planning and land management policy by the Zhangye City government.

**Table 2:** Scenario design based on critical indicators in the study area. ‘S1’ is the historical land-use demand scenario reflecting land demand growth as a continuation of the historical period. ‘S2’ is the moderate protection scenario in which land demand growth was limited by setting critical indicators of economic and social development. ‘S3’ is the strict protection scenario in which land demand growth was simulated with multiple strict protections in the study area.

Critical indicator	2000–2009	S1	S2	S3
Growth rate of GDP (%)	18.13	20	16	12
Growth rate of natural population (‰)	6.73	8	6	4
Urbanization (%)	29.26	40	30	20
Food self-sufficiency rate (%)	95	110	100	90
Growth rate of food production (%)	1	1.3	1	0.7

The scenarios considered in this paper provide a reference guide for local governments as well as for other stakeholders involved in regional development, helping them understand the outcomes of the various development policies and potential modeling approaches presented in this study. Finally, the method was used to simulate different scenarios with multiple critical indicator values (Table 2) in the SD-CLUE-S model. The indicators included the 33 parameters that affected land demand growth, as well as assigning various demand values in the spatial policy module and comparing them with land-supply figures provided by the CLUE-S module. Finally, a spatialization simulation was carried out using the quantitative relationship of land supply and scenario demand.

### Carbon storage model

The InVEST carbon storage module uses a simplified carbon cycle that quantifies the amount of static carbon storage and dynamic sequestration or loss based on five basic carbon density pools: aboveground biomass, belowground biomass, soil, dead organic matter, and harvested wood products (HWP) [36]. The carbon density of each land-use type and a land-use/land-cover map were used as the primary input data to estimate carbon storage in each grid cell. Only four carbon pools were considered in this study because of the limited number of measurements of HWP data. These included aboveground biomass (*Ca*), belowground biomass (*Cb*), soil organic carbon (*Cs*), and dead organic matter (*Cd*). The carbon in each pool was then aggregated over different land-use types to estimate carbon storage across the landscape. The carbon storage *S*_*m*,*i*,*j*_ for a given grid cell *(i*,*j)* with land-use type *m* can be calculated as:

$$C_{m,i,j}=A\times \left(Ca_{m,i,j}+Cb_{m,i,j}+Cs_{m,i,j}+Cd_{m,i,j}\right)$$ (2)

where *A* is the actual area of each grid cell (ha) and *Ca*_*m*,*i*,*j*_, *Cb*_*m*,*i*,*j*_, *Cs*_*m*,*i*,*j*_, and *Cd*_*m*,*i*,*j*_ are the aboveground carbon density (MgC·ha^−1^), belowground carbon density (MgC·ha^−1^), soil organic carbon density (MgC·ha^−1^), and dead organic matter carbon density (MgC·ha^−1^) for grid cell *(i*,*j)* with land-use type *m*. Hence, carbon storage *C* and sequestration *S* across the whole region can be calculated as:

$$C=\overset{n}{\underset{m=1}{\sum }}C_{m,i,j}$$ (3)

$$S=C_{}^{T2}-C_{}^{T1}$$ (4)

where *C*^*T*2^ and *C*^*T*1^ indicate static carbon storage in years *T2* and *T1* (*T2* > *T1*) respectively. Different kinds of information on the four carbon pools in the study were collected by field experiments and literature review (Table 3). Finally, carbon storage was calculated in woodland, cropland, grassland, built-up land, and unused land respectively. The carbon in water was assumed to be 0. The carbon-pool data used in this research were the only available data based on the specific land-use types in the oasis, and the carbon density of each pool was assumed not to have changed during the historical period (2000–2009) and the scenario period.

**Table 3:** Carbon pools of different land-use types in InVEST (units: MgC•ha^−1^). ‘*Ca*’ refers to the aboveground biomass. ‘*Cb*’ refers to the belowground biomass. ‘*Cs*’ refers to the soil organic carbon. ‘*Cd*’ refers to the dead organic matter.

Land-use type	*Ca*	*Cb*	*Cs*	*Cd*	Sources
Cropland	3	2	873.33	1.5	[11,36,37]
Woodland	36.05	22.5	1050.75	11.75	[11,36,37]
Grassland	1.8	1.43	656.33	0.22	[11,36,37]
Built-up land	0	0	388.67	0	[36]
Unused land	0.4	0.83	245	0	[11,36,37]

**Fig 3:**
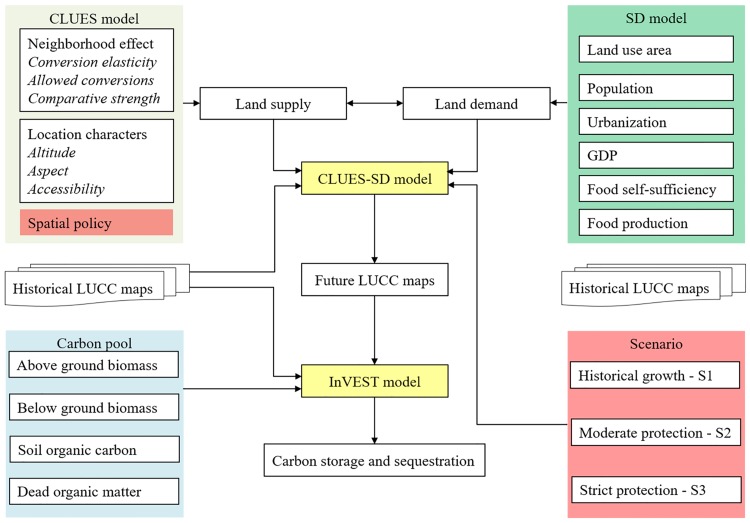
General modeling framework of the integrated SD-CLUE-S and InVEST model.

The general modeling framework is shown in Fig 3. The land-use/land-cover module was used by the SD-CLUE-S model, and the carbon storage module was used by InVEST. Future land-use changes in the study area from 2009 to 2018 were simulated using the calibrated SD-CLUE-S model. Finally, the potential impacts of scenario-based land-use change on oasis carbon storage were simulated by the InVEST model.

## Results

### Simulation of land-use change

**Fig 4:**
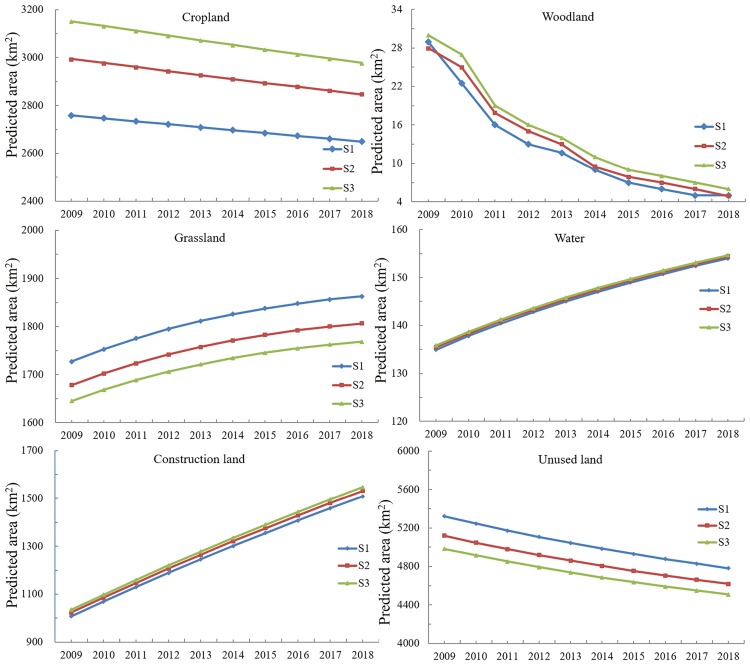
Predicted land demand area in the oasis under three scenarios from 2009 to 2018 (units: km^2^).

**Fig 5:**
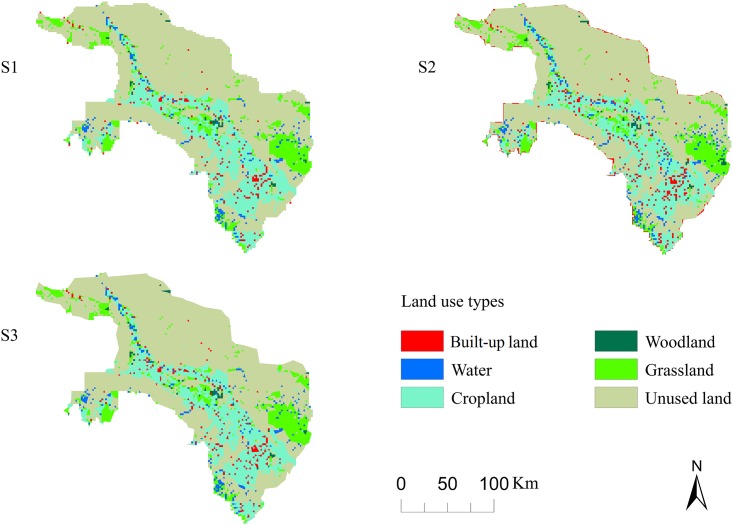
Simulations of oasis land use in 2018 under scenarios S1, S2, and S3.

A significant feature of the SD-CLUE-S model was its ability to analyze spatio-temporal land-use change based on demand projected by regional development plans. The rate of land-use change and its spatial distribution were affected by different land demand scenarios (Figs 4 and 5). The core area of oasis land (including cropland, woodland, grassland, built-up land, and water) increased by 12.5%, from 4008 km^2^ in 2000 to 4509 km^2^ in 2009. The results obtained with scenario S1 showed that the area of built-up land expanded by 49.75% from 2009 to 2018. This scenario also showed that these high expansion rates caused a decrease in unused land and a degradation of cropland. The results of scenario S3 showed a smaller increase in built-up land compared with S1 and S2. The area of built-up land expanded by 45.51% from 2009 to 2018 in scenario S3. Furthermore, note that all the area expansion of built-up land under the three scenarios will largely occur in the center of the oasis (Fig 5). These findings show that scenario S3 saves the largest area of land resources under strict protection measures for sustainable oasis development compared with the results of S1 and S2.

### Simulation of carbon storage

**Fig 6:**
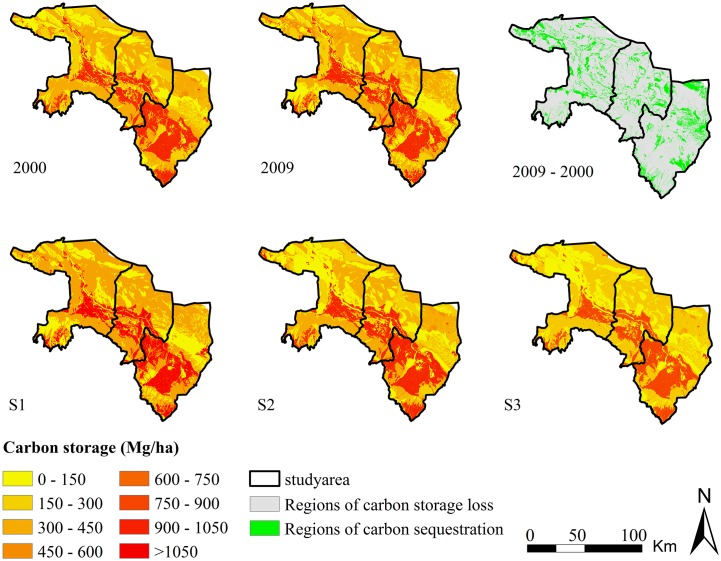
Carbon storage in the oasis for 2000, 2009, and 2018 under different scenarios.

The results based on the InVEST model showed that oasis carbon storage increased from 374.72×10^6^ Mg in 2000 to 377.75×10^6^ Mg in 2009, with an average annual sequestration of 3.03×10^5^ Mg and a corresponding sequestration rate of 0.81% (Table 4). During this period, expansion of oasis land has resulted in significant carbon sequestration at pixel scale in pockets that are numerous and scattered across the oasis (Fig 6).

**Table 4:** Oasis carbon storage based on InVEST and land-use change in 2000, 2009, S1, S2, and S3.

Carbon storage context	Mean (Mg/ha)	Total (10^6^ Mg)
2000	354.08	374.72
2009	356.94	377.75
S1	353.61	374.22
S2	354.23	374.89
S3	354.51	375.18

In 2009–2018, scenario S1 showed rapid land-use change caused by human activities. Compared with the baseline in 2009, oasis carbon storage with S1 is expected to decrease by 3.53×10^6^ Mg from 2009 to 2018, with an average annual carbon loss of 3.53×10^5^ Mg and a corresponding loss rate of 0.93%. Carbon storage loss will occur mainly in cropland and woodland. The results obtained from scenarios S2 and S3 showed that moderate and strict protection resulted in smaller disturbances by human activities. Under S2, carbon storage will decrease by 2.87×10^6^ Mg from 2009 to 2018, with an average annual carbon loss of 2.87×10^5^ Mg and a corresponding loss rate of 0.76%. Carbon storage with S3 will decrease by 2.58×10^6^ Mg from 2009 to 2018, with an average annual carbon loss of 2.58×10^5^ Mg and a corresponding loss rate of 0.68%. Unlike S1, most carbon sequestration occurred in reserved wetlands and productive agricultural areas, which are located mainly in the central parts of the oasis.

The simulation results showed that scenario S3 saved the largest amounts of stored carbon under a strict protective socioeconomic development scenario for the Zhangye oasis compared with scenarios S1 and S2. The results with S3 also indicated that a compact land-use form can facilitate provision of carbon storage by oasis managers. Compared with S1 and S2, S3 is the preferred regional development form for this oasis.

## Discussion

Integrating InVEST and a land-use model has shown prospective applicability in assessing the potential impacts of regional land-use change on multiple ecosystem services and in decision-making about tradeoffs in an oasis environment. In the case of the SD-CLUE-S model, the various spatial resolutions of input data were first resampled uniformly to 100 m, and the temporal scale of the output results were also noted during modeling. Using a decadal input dataset, a consistent and spatially explicit oasis land-use dataset was generated based on the model. However, more study is needed to determine the potential impact of using a shorter time series. For example, yearly input data may actually generate better simulations. Second, the land-use datasets in 2000–2009 were independent and were used in different parts of the calibration, validation, and future prediction processes. These input data and simulations covered a specific period when dramatic land-use changes were occurring within the Zhangye oasis. Detailed information on the rest of the model application is available in reference [22]. Third, due to rapid population growth, regional development has been under heavy pressure in the oasis since the beginning of the 1990s [29]. Based on the results of the change analysis, the expansion of built-up land occurred largely at the expense of cropland in 2000–2009, and the actual growth was particularly significant on both sides of rivers and reservoirs across the oasis. Finally, the definition of the land-conversion coefficient in the SD-CLUE-S model is determined by the modeler’s knowledge gained in the study area; more research into parameter experiments is needed to understand the impact on simulation of oasis land-use change.

In addition, the simulations demonstrated the usefulness of scenario-based methods in predicting possible changes in future oasis carbon storage services. It is important to understand how socioeconomic development and land demand respond to different scenarios and thereby to help land management and environmental planning with research-based statements. An increase or reduction in the level of oasis land development was found to be related to land-use distribution and carbon storage. Nevertheless, uncertainties exist regarding the magnitude of scenario indicators (Table 2) generated from local statistical data and socioeconomic development plans. In fact, socioeconomic indicators are given a higher priority than other driving forces in influencing future land-use patterns in most land-use models [38]. Furthermore, to strengthen local decision-making by providing strict protection under land demand growth and minimizing adverse impacts on ecosystem services, payments for carbon storage services should be implemented in the Zhangye oasis so that downstream beneficiaries and other oasis service stakeholders would contribute to sustainable oasis management and development. Although payments have not yet been implemented in the study area, the current study, which provides a preliminary background for land-use patterns and ecosystem services, can assist the local government to develop the optimal practical mechanism for assessing and collecting payments from related stakeholders to protect the oasis eco-environment and to facilitate provision of ecosystem services.

The InVEST model was designed to quantify multiple ecosystem services provided by nature [35]. Besides carbon storage, other crucial ecosystem services (e.g., ecotourism, biodiversity, food production, and sediment retention) would be affected by land-use changes in a desert oasis. Changes in multiple ecosystem services may yield local tradeoffs and synergies [16]. Quantifying and mapping ecosystem services affected by land use under the driving forces of human activities will be the basic way to identify and reduce the negative effects of land degradation on the fragile ecological environment of the Zhangye oasis. In addition, the role of different stakeholders’ involvement in decision-making is important in defining different land management scenarios [39].

## Conclusions

This study presents an integrated modeling method combining SD-CLUE-S and InVEST models to assess the impacts of land-use changes on oasis carbon storage in Northwest China. The results showed that various marginal changes in pixels and their regional carbon storage were related to multiple land-use changes under the driving forces of human activities. The effects of built-up land expansion were especially notable, with the resulting significant decrease in cropland. Intensifying land-use demand and limited supply under extreme socioeconomic development scenarios suggest very significant changes in oasis carbon storage. These results have emphasized the scientific value of landscape ecological research in showing the relationships between the effects of different land demand patterns, land-use patterns, and their consequences for ecosystem services at the oasis level. Furthermore, the results can be used to help government managers encourage local stakeholders and beneficiaries of upstream and downstream ecological product users to contribute funds and strategies for facilitating landscape patterns and ecological processes in Zhangye oasis by implementing two potential land conservation activities—protection of cropland and control of rapid built-up land expansion.

## Supporting information

S1 Table Areas of initial land-use types. (PDF)
