# Supplementary material for: Integrating the SD-CLUE-S and InVEST models into assessment of oasis carbon storage in northwestern China
Source: PLoS One. 2017 Feb 23;12(2):e0172494. doi: 10.1371/journal.pone.0172494 (PMC5322964; doi:10.1371/journal.pone.0172494)
Supplement: S1 Table — (PDF) [file pone.0172494.s001.pdf]

Integrating the SD-CLUE-S and InVEST models into assessment of oasis carbon storage in northwestern China

Youjia Liang, Lijun Liu, Jiejun Huang

S1 Table 1. Areas of initial land-use types.

Initial land-use types	coding	Area (2000, km^2)	Area (2005, km^2)	Area (2009, km^2)
Forest	21	11.81	21.48	30.95
Spinney	22	33.76	37.325	41.09
Open woodland	23	37.91	54.705	71.9
Other woodland	24	1.21	6.295	12.58
High coverage grassland	31	21.17	150.35	278.93
Middle coverage grassland	32	207.56	281.6	355.44
Low coverage grassland	33	933.77	1291.365	1648.36
Canal	41	125.58	89.085	52.79
Lake	42	1.45	2.21	2.57
Glaciers and permanent snow	43	31.11	24.635	18.56
Shallow	46	186.86	165.165	143.87
Urban areas	51	13.39	13.78	14.57
Rural areas	52	115.1	119.665	124.43
Other construction areas	53	14.02	16.42	18.82
Unused land	61	7287.46	7038.43	6790.4
Paddy field	111	0.01	0.025	0.04
Mountain cropland	121	22.89	12.43	1.57
Plain cropland	123	2250.71	1970.925	1693.14
